# MP2-Based Correction Scheme to Approach the Limit of a Complete Pair Natural Orbitals Space in DLPNO-CCSD(T) Calculations

**DOI:** 10.1021/acs.jctc.3c00444

**Published:** 2023-06-20

**Authors:** James Pogrebetsky, Alexandra Siklitskaya, Adam Kubas

**Affiliations:** Institute of Physical Chemistry, Polish Academy of Sciences, Kasprzaka 44/52, Warszawa 01-224, Poland

## Abstract

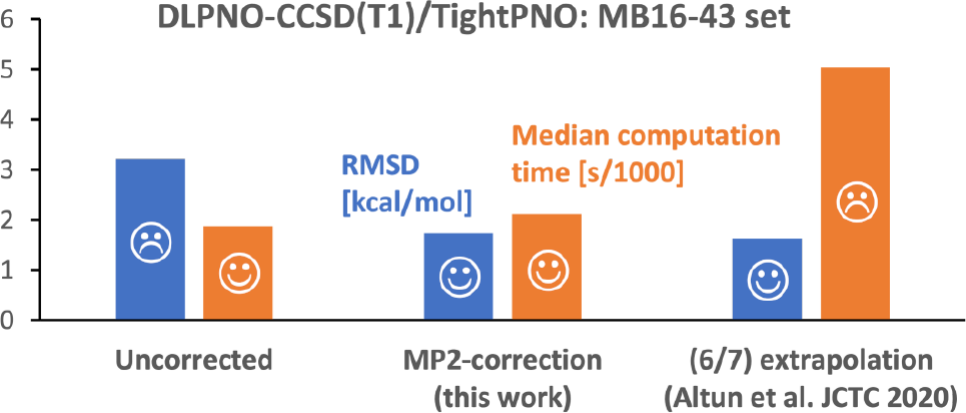

The domain-based local pair natural orbital (PNO) coupled-cluster DLPNO-CCSD(T) method has been proven to provide accurate single-point energies at a fraction of the cost of canonical CCSD(T) calculations. However, the desired “chemical accuracy” can only be obtained with a large PNO space and extended basis set. We present a simple yet accurate and efficient correction scheme based on a perturbative approach. Here, in addition to DLPNO-CCSD(T) energy, one calculates DLPNO-MP2 correlation energy with the same settings as in the preceding coupled-cluster calculation. In the next step, the canonical MP2 correlation energy is obtained in the same orbital basis. This can be efficiently performed for essentially all molecule sizes accessible with the DLPNO-CCSD(T) method. By taking the difference between the canonical MP2 and DLPNO-MP2 energies, we obtain a correction term that can be added to the DLPNO-CCSD(T) correlation energy. This way, one can obtain the total correlation energy close to the limit of the complete PNO space (cPNO). The presented approach allows us to significantly increase the accuracy of the DLPNO-CCSD(T) method for both closed- and open-shell systems. The latter are known to be especially challenging for locally correlated methods. Unlike the previously developed PNO extrapolation procedure by Altun, Neese, and Bistoni (J. Chem. Theory Comput.2020, 16, 6142−61493289771210.1021/acs.jctc.0c00344PMC7586325), this strategy allows us to get the DLPNO-CCSD(T) correlation energy at the cPNO limit in a cost-efficient way, resulting in a minimal overall increase in calculation time as compared to the uncorrected method.

## 1 Introduction

The struggle for higher accuracy is a never-ending battle in the field of computational studies. The right answer is in principle known and is called the full configuration interaction (FCI) method, but it is limited to very small molecules. In an attempt to build a hierarchy of cheaper methods approaching FCI accuracy, one typically starts with the Hartree–Fock (HF) method. Subsequently, a new correlated wave function is constructed by allowing certain excitations out of a HF determinant in order to recover FCI correlation energy. However, the cost of the calculations increases exponentially with the allowed order of excitations.

A simple remedy to make the calculations more accessible is to cut a part of the orbital space, leaving only the most important orbitals. While truncating canonical HF orbitals may lead to uncontrolled issues, natural orbitals (NOs) are better suited for this purpose. Such orbitals proposed by Löwdin^1^ provide a good basis for rapidly converging CI expansion. Moreover, the CI calculations can then be performed in such a truncated space spanned by strongly and weakly occupied NOs. However, since some untruncated CI calculations are essential to obtain good NOs, this approach is not well-suited for large systems. A more convenient pair-natural orbital (PNO) approximation (sometimes called pseudonatural orbitals) has been proposed and explored by Edmiston and Krauss,^2^ by Meyer,^3,4^ and later by Ahlrichs and co-workers.^5^ At early stages, PNOs have been used with an independent electron pair approximation (IEPA),^4,6^ coupled electron pair approximation (CEPA), or configuration interaction doubles (CID).^4,5^

These early implementations have been able to produce a decent agreement with the experimental data.^7,8^ However, the merit of the PNO approach only came after the development of more accurate methods such as the coupled cluster theory (CC).^9−12^ This new combination turned very accurate and expensive calculations into robust wave function based alternatives to the density functional theory calculations with its approximated density functionals, where the accuracy is hard to control.^13,14^

Nowadays, coupled cluster method with single and double excitations and perturbative treatment of triple excitations [CCSD(T)] is considered a “gold standard” since it can provide accuracy of less than 1 kcal/mol.^15,16^ Of course, such high accuracy can only be achieved in single-reference cases and at the complete basis set (CBS) limit. Therefore, real-life applications of CCSD(T) are rather limited to small molecules. The scope of applications can be significantly extended by exploring the locality of electron correlation,^17−20^ as well as compression of the virtual orbital space via PNOs. Some prominent developments include works of Werner and Schütz,^20−27^ Head-Gordon,^28,29^ and Neese.^30−34^ Present work focuses on the domain-based local pair natural orbital coupled-cluster (DLPNO-CCSD(T)) method developed by Neese’s group.

DLPNO-CCSD(T) method combines the local correlation theories with the PNO machinery.^35^ It relies on the fact that most PNOs are rather localized, and when combined with localized internal orbitals, it allows for a very compact expansion of the wave function.^34^ The method has been implemented both for closed- and open-shell systems and has been shown to scale favorably with the system size.^35,36^

Several groups have demonstrated that the method produces results of “chemical accuracy” level.^37−39^ On the hand, Liakos et al.^38^ have noted that the accuracy of the DLPNO-CCSD(T) is lower for open-shell systems. The latter have been found to be more demanding in terms of PNO space size. This is not surprising as already Meyer^4^ had noted that certain electron delocalization inevitably occurs in open-shell molecules. Moreover, the realization of the triples (T) correction significantly influences the results. The noniterative, original scheme, denoted here as (T0),^33^ does not produce proper spin-state energetics in open-shell systems.^40,41^ This may be improved by employing iterative triples correction, denoted here as (T1).^42−44^

Decreasing the truncation parameters for PNO occupation numbers provides a systematic way to converge the results to canonical CCSD(T) numbers. At the same time, however, the computational time increases along with memory consumption. Altun and co-workers^45^ have observed that the DLPNO-CCSD(T) correlation energy converges exponentially with the increasing number of PNOs. Based on this observation, they have developed a two-point extrapolation procedure that greatly increases accuracy of the calculation. Still, the necessity to perform DLPNO-CCSD(T) calculations twice with different parameters makes this approach expensive in terms of computational time. As a modification of this procedure Drosou et al.^44^ proposed to use lower truncation parameters for the extrapolation without significant decrease of accuracy but with improved efficiency. One should note that the entire procedure involves fitted parameter *F* that depends on the actual PNO truncation parameters used in the extrapolation.

It is interesting to note that the two-point extrapolation is a well-known technique to reach the complete basis set (CBS) limit in the wave function based calculations.^46,47^ Another, more economic way to approximate the CBS of a given high-level method, pioneered by Hobza and co-workers,^48−50^ is to calculate the MP2 energy with the same basis set and then estimate the MP2-CBS energy. By taking the difference between the MP2-CBS and finite-basis MP2 correlation energies, one obtains a correction factor that can be added to the high-level method correlation energy calculated at the finite basis set. In the context of locally correlated methods, Wang and co-workers used this method to reach the CBS limit in their generalized energy-based fragmentation CCSD(T)-F12a method.^51^ Inspired by this approach, we have designed an analogous correction scheme to approximate the complete PNO space of the DLPNO-CCSD(T). Here, after the DLPNO-CCSD(T) energy evaluation, one calculates the DLPNO-MP2 correlation energy with the same PNO settings. In the next step, the canonical MP2 correlation energy is obtained at the same basis set. This can be efficiently performed for essentially all molecule sizes accessible with the DLPNO-CCSD(T) method. By taking the difference between canonical MP2 (*E*_corr_^MP2^) and DLPNO-MP2 (*E*_corr_^DLPNO-MP2^) correlation energies, one can obtain a correction term that can be added to the DLPNO-CCSD(T) correlation energy (*E*_corr_^DLPNO-CCSD(T)^). This way, one can obtain total correlation energy close to the limit of the complete PNO space (denoted here as cDLPNO-CCSD(T), *E*_corr_^cDLPNO-CCSD(T)^) at a given basis set without any fitted parameters:
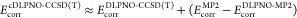
1

In this work, we first evaluate the approach against two subsets of the GMTKN55 database:^52^ S22 and MB16-43. The former constitutes a classical test to check the accuracy of the quantum-chemical method to describe noncovalent interactions.^53^ It contains small model complexes, DNA base pairs, and amino acid pairs. The MB16-43 contains an interesting set of decomposition reactions of nonexistent but virtually stable molecules. Most importantly, it involves open-shell systems and has been shown to be the most challenging for the DLPNO-CCSD(T) calculations reported to date.^38,45^ Using computational timings obtained for the MB16-43, we also demonstrate the efficiency of the proposed scheme.

As our main application field of the DLPNO-CCSD(T) approach is transition metal catalyzed reactions, we checked our correction scheme against the modified MOBH35 set^54,55^ that focuses on metal–organic barrier heights for relatively large closed-shell complexes. To explore the accuracy of our approach for open-shell transition metal-containing systems, we designed an additional benchmark. Here, we test the method against the canonical CCSD(T) for a set of potential energy surface scans for the six three-atomic systems, featuring a metal–carbon monoxide interaction in neutral and cationic forms. These are the smallest models for CO reactivity toward single-atom catalysts.^56−58^ The size of these systems permits exploration of basis-set dependency of the PNO space incompleteness error and consequently the behavior of the proposed correction scheme with respect to the basis set. We discuss our results in light of previous investigations that look into basis set-dependency of the DLPNO-CCSD(T) results obtained with various PNO truncation parameters.^37,59^

## 2 Computational Details

All calculations have been conducted with the use of ORCA 5.0.2 package.^60^ Reference canonical coupled-cluster calculations have been performed with unrestricted reference (UHF) and quasi-restricted open-shell orbitals (QROs).^61^ DLPNO-CCSD(T) correlation energies have been evaluated with the UHF reference as well, except for entries of MOBH35. In the latter case, due to efficiency reasons, restricted Hartree–Fock reference was employed with disabled full MP2 guess to stay consistent with UHF-based calculations. Single-point energies for S22 and MB16-43 test sets have been evaluated using the scalar relativistic second-order Douglas–Kroll–Hess Hamiltonian and the compatible aug-cc-pVDZ-DK basis set.^62^ The resolution-of-identity (RI)^63^ auxiliary basis set has been generated automatically with the AutoAUX feature.^64^ This setup is the same as in the work of Altun and co-workers;^45^ therefore results and timings are comparable. Ahlrichs’s split-valence Def2-SVP, Def2-TZVP, Def2-TZVPP, and Def2-QZVPP basis sets^65^ have been used for other calculations (see details in text) along with the corresponding auxiliary basis sets.^66,67^ Def2 effective core potentials (ECPs) have been employed in all calculations involving late transition elements (Pd, Pt, Au).^68^ We have also made use of the RI-MP2 approach for canonical MP2 calculations.^69^

Recently, Bistoni and co-workers^45^ have developed a DLPNO extrapolation procedure to which the presented scheme may be compared. Inspired by the two-point basis set extrapolation schemes, they proposed to approach the complete PNO space with extrapolated correlation energy values obtained in two calculations involving a different number of PNOs, controlled by the *T*_cutPNO_ parameter. Although this approach greatly increases the accuracy of the calculation, the same can be said about the computational cost. The extrapolation from *T*_cutPNO_ of 10^–*x*^ and 10^–*y*^ is denoted here as (*x*/*y*) extrapolation. Other truncation parameters are set according to the TightPNO keyword in the ORCA package (see Table 1). As found by Altun et al.,^45^ the increase of *x* and *y* brings the DLPNO-CCSD(T1) correlation energy to the proximity of the canonical CCSD(T) method.

**Table 1:** Critical DLPNO Settings^a^ Associated with Simple Keywords in the ORCA Nomenclature Used in the Proposed Correction Scheme

keyword	*T*_cutPNO_	*T*_cutMKN_	*T*_cutDO_
NormalPNO	3.33 × 10^–7^	10^–3^	1.0 × 10^–2^
TightPNO	1.00 × 10^–7^	10^–3^	5.0 × 10^–3^

a *T*_cutPNO_, *T*_cutPAIR_, *T*_cutDO_. *T*_cutDO_ has been used only for the DLPNO-MP2 calculations and closed-shell DLPNO-CCSD(T).

In the proposed scheme, we have made use of two sets of the default DLPNO-CCSD(T) settings: NormalPNO and TightPNO (Table 1). These have been applied consistently with both DLPNO-CCSD(T) and DLPNO-MP2 methods. More specifically, the DLPNO-CCSD(T) calculations have been performed by setting NormalPNO or TightPNO in the simple input line while the truncation parameters for the DLPNO-MP2 calculations have been defined explicitly in the *%mp*2 block. The Supporting Information contains an example compound job that can be easily used with the current ORCA version to obtain DLPNO-CCSD(T) energies corrected with the proposed scheme.

One should note that PNOs in open-shell DLPNO-CCSD(T) and DLPNO-MP2 calculations are constructed in a different way. In the former case, the high-spin open-shell variant of the *N*-electron valence perturbation theory formalism is used to define the initial guess wave function and consequently also the open-shell PNOs.^36^ The DLPNO-MP2 follows the genuine UHF-MP2 implementation, i.e., without referencing to QROs. Here, the critical point is to obtain the PNO correction factor from the DLPNO-MP2 and the canonical MP2 with the same reference determinant and with the same MP2 implementation. In principle, one can use QROs generated in a separate step for UHF-MP2/UHF-DLPNO-MP2 calculations. However, we did not find this beneficial as the resulting interaction curves for the model Pt–CO system were virtually identical irrespective of the tested reference (see SI Figure S1). Additionally, to confirm the similarity of the PNO spaces in DLPNO-CCSD(T) and DLPNO-MP2 calculations, we checked the correlation between semilocal MP2 energy in the NEV-PNO space of the DLPNO-CCSD(T) calculations and the DLPNO-MP2 correlation energy. This came out to be linear (see SI Figure S2), which means that both PNO spaces cover the same physical interactions.

The current work shows that even with different strategies employed for the PNO construction in the DLPNO-CCSD(T) and the DLPNO-MP2 methods, the final correction factor provides significant improvement to the uncorrected DLPNO-CCSD(T) method.

Reference CCSD(T) data for the S22 and MB16-43 sets has been taken from the work of Altun et al.^45^ while the modified MOBH35 has been referenced to the CCSD(T)/def2-SVP values obtained by Semidalas and Martin^55^ with the PSI4 code. Statistical errors have been evaluated using the following error measures:

- mean error, ME = (∑_*i*=1_^*i*=*N*^(*y*_*i*_ – *y*_*i*_^ref^))/*N*
- mean absolute error, MAE = (∑_*i*=1_^*i*=*N*^|*y*_*i*_ – *y*_*i*_^ref^|)/*N*
- root-mean-square error,
- most positive error, MPE = max_*i*=1_^*i*=*N*^(*y*_*i*_ – *y*_*i*_^ref^)
- most negative error, MNE = min_*i*=1_^*i*=*N*^(*y*_*i*_ – *y*_*i*_^ref^)
- error spread, ES = MPE – MNE

where *y*_*i*_^ref^ is a reference value for the *i*th entry estimated with a calculated value *y*_*i*_ and *N* is the number of entries in the database.

All timings reported in the study have been obtained with dedicated computational nodes equipped with 18-core Intel Xeon Gold 6254 CPU running at 3.10 GHz, 128 Gb of RAM, and SSD storage. The calculations have been run with 6 cores and 5 Gb RAM memory available per core.

## 3 Results and Discussions

### 3.1 S22 Test Set

In the first step, the correction proposed in eq 1 was tested against the S22 set composed of 22 interaction energies between small, closed-shell organic molecules. The calculations for this benchmark database constitute a kind of sanity test as any correction scheme designed to approach complete PNO space should improve the interaction energies of these dimers.

Statistical measures for the uncorrected (DLPNO) and the corrected (cDLPNO) calculations performed with NormalPNO and TightPNO settings are presented in the Table 2 The perturbative triples have been evaluated either with the original noniterative scheme (T0) or with the iterative (T1) method. For comparison, we also present statistics for the recently reported extrapolation scheme by Altun et al.^45^

**Table 2:** Correlation Energies Error Evaluation [in kcal/mol] of the Original (DLPNO) and Corrected (cDLPNO) DLPNO-CCSD(T0/T1) Methods against the Canonical CCSD(T) Energies Obtained for the S22 Set^45^^a^

	NormalPNO				TightPNO					
	T0		T1		T0		T1			
	DLPNO	cDLPNO	DLPNO	cDLPNO	DLPNO	cDLPNO	DLPNO	cDLPNO	Extr.(5/6)	Extr.(6/7)
ME	–0.089	0.128	–0.079	0.138	–0.310	–0.171	–0.263	–0.124	0.141	–0.041
MAE	0.430	0.395	0.452	0.419	0.429	0.172	0.387	0.133	0.213	0.173
RMSE	0.619	0.552	0.652	0.588	0.572	0.237	0.541	0.196	0.409	0.350
MPE	1.262	1.419	1.274	1.538	1.312	0.015	1.361	0.061	1.648	1.416
MNE	–1.413	–0.669	–1.519	–0.697	–1.201	–0.587	–1.254	–0.473	–0.490	–0.693
ES	2.675	2.088	2.793	2.234	2.513	0.602	2.615	0.534	2.138	2.109

a Extrapolated (5/6) and (6/7) T1 values have been taken from the literature and are provided in the last two columns.^45^ Error measures are explained in the Computation Details section.

We have observed clear accuracy improvement when the correction for the complete PNO space is accounted for. Interestingly, already with economic settings (NormalPNO and (T0) triples) the RMSE is below 1 kcal/mol. However, to bring MPE and MNE below 1 kcal/mol, it is important to use the TightPNO settings. Our approach works consistently well irrespective of perturbative triples treatment as expected for closed-shell molecules. Among the proposed setups, cDLPNO-CCSD(T) with TightPNO thresholds outperforms extrapolated results, especially in terms of error spread that drops below 0.6 kcal/mol in our case as compared to >2 kcal/mol for the extrapolated values.^45^

### 3.2 MB16-43 Subset of the GMTKN55 Database

The MB16-43 has been the most challenging set in the recent benchmark studies of both the original^38^ and extrapolated DLPNO-CCSD(T) methods.^45^ This is the case because of the presence of open-shell species in the set that require more extended PNO space to recover the canonical correlation energy. Due to the size and nature of the molecules in the set (relatively large polyatomic species including molecules of closed- and open-shell character composed of atoms up to the third period), this database is also well suited for timing comparison.

According to Table 3, the proposed correction scheme allows for significant improvement of the obtained results. Here, the combination of TightPNO settings and iterative treatment of the triple excitations (T1) provides results very close to extrapolated (6/7) values of Altun et al.^45^ (RMSE of 1.629 kcal/mol compared to 1.634 kcal/mol, respectively). For both methods, MAE is virtually identical (ca. 1.4 kcal/mol), but cDLPNO provides slightly larger error spread (ES of 3.560 kcal/mol vs 2.889 kcal/mol). The latter could be traced back to the fact that the MP2 is not the optimal choice for open-shell systems due to unbalanced treatment of same-spin and opposite-spin correlation contributions as stated initially by Grimme^70^ and shown rigorously later by Fink.^71^ One should note, however, that our cDLPNO method even with the tightest settings (TightPNO with T1) is about 2.5 times faster than the alternative (6/7) extrapolation technique (see last entry in Table 3).

**Table 3:** Correlation Energies Error Evaluation [in kcal/mol] of the Original (DLPNO) and Corrected (cDLPNO) DLPNO-CCSD(T0/T1) Methods against the Canonical CCSD(T) Energies Obtained for the MB16-43 Set^45^^a^

	NormalPNO				TightPNO					
	T0		T1		T0		T1			
	DLPNO	cDLPNO	DLPNO	cDLPNO	DLPNO	cDLPNO	DLPNO	cDLPNO	Extr.(5/6)^45^	Extr.(6/7)^45^
ME	–5.277	–2.915	–4.269	–1.899	–4.207	–2.630	–3.057	–1.416	–3.191‘	–1.476
MAE	5.277	2.915	4.269	1.899	4.207	2.630	3.057	1.441	3.191	1.476
RMSE	5.624	3.141	4.526	2.181	4.487	2.831	3.240	1.629	3.411	1.634
MPE	–1.766	–0.603	–1.291	–0.069	–1.474	–0.618	–0.964	0.530	–0.780	–0.265
MNE	–9.713	–6.363	–7.872	–4.619	–7.888	–5.804	–5.679	–3.031	–5.580	–3.153
ES	7.947	5.759	6.581	4.550	6.414	5.186	4.715	3.560	4.800	2.889
median computational time [s]	292	502	701	930	811	1006	1876	2116	1393	5044

a Extrapolated (5/6) and (6/7) values have been taken from the literature and are provided in the last two columns.^45^ Error measures are explained in the Computation Details section. Median computational times for all 43 large molecules in the set are provided in the last entry for each method.

For the systems in the MB16-43 set, the (T1) iterative scheme for the perturbative triple excitation is clearly favored over noniterative original (T0) formulation. Interestingly, by keeping the (T1) option and loosening the PNO parameters to NormalPNO, one obtains cDLPNO errors smaller than for the extrapolation scheme (5/6) about 30% faster.

### 3.3 MOBH35 Database

The original MOBH35 database of Iron and Janes^54^ contains 35 single-step reactions (substrate, transition state, and product) of relatively large, closed-shell transition metal-containing systems. Following the analysis by Semidalas and Martin,^55^ we decided to remove entries 8 and 9 due to severe and unbalanced static correlation that renders any single-reference coupled-cluster calculations unreliable. At the same time, the authors provide CCSD(T)/def2-SVP reference values for the entire MOBH35 set except entries 17–20 that are too large for the computations. Thus, this study considers 29 organometallic reactions.

The overall performance of the proposed correction scheme is satisfying, providing accuracy similar to that of the previously proposed scheme at a significantly lower computational cost. For example, the combination of TightPNO settings and the (T1) approach provides results very close to the extrapolated (6/7) values (RMSE of 0.686 kcal/mol compared to 0.676 kcal/mol, respectively; Table 4).

**Table 4:** Total Energies Evaluation [in kcal/mol] of the Original (DLPNO) and Corrected (cDLPNO) DLPNO-CCSD(T0/T1) Methods against the Canonical CCSD(T) Energies Obtained for the Modified MOBH35 Set^55^^a^

	NormalPNO				TightPNO					
	T0		T1		T0		T1			
	DLPNO	cDLPNO	DLPNO	cDLPNO	DLPNO	cDLPNO	DLPNO	cDLPNO	Extr.(5/6)	Extr.(6/7)
ME	0.076	–0.048	0.050	–0.07	0.022	–0.035	–0.011	–0.068	–0.073	–0.153
MAE	1.029	0.868	0.910	0.773	0.786	0.686	0.614	0.506	0.880	0.496
RMSE	1.445	1.174	1.246	1.046	1.089	0.936	0.831	0.686	1.093	0.676
MPE	3.974	2.910	3.707	2.643	2.716	2.056	2.267	1.666	3.279	1.684
MNE	–5.115	–4.820	–3.859	–3.564	–3.556	–3.346	–2.227	–2.017	–2.374	–2.049
ES	9.089	7.730	7.566	6.207	6.272	5.402	4.494	3.683	5.653	3.733

a Extrapolated (5/6) and (6/7) T1 values have been obtained according to the method of Altun and co-workers.^45^ Error measures are explained in the Computation Details section.

Relatively large MPE and MNE for the corrected and uncorrected results highlight a deeper issue with the adapted default DLPNO-CCSD(T) method. First of all, the iterative T1 scheme does not account sufficiently for the dynamic correlation-induced orbital relaxation effects as has been demonstrated very recently by Altun et al.^72^ Semidalas and Martin^55^ have shown that the discrepancy of the DLPNO-CCSD(T1) with the canonical CCSD(T) can be related to the energy difference between (T1) and (T0) triples corrections. An empirical correction (denoted as DECIOR) has later been proposed^72^ for MOBH35 as the DLPNO-CCSD(T1) error shows a roughly linear correlation with square of the norm of the single-amplitude vector, ∥*t*_1_∥^2^. Another source of errors in the DLPNO-CCSD(T) calculations is the treatment of semicore–valence correlations, which have been shown to be more demanding in terms of the *T*_cutPNO_. However, tightening of the truncation parameter for the PNOs involving semicore orbitals (e.g., 3s3p for 3d transition metals) is not automatic. It requires redefinition of the number of core electrons for relevant centers as well as adjustment of the energetic window for the frozen core treatment. In principle both corrections can be applied with the current scheme, but we prefer to keep the defaults that can be adjusted for particular applications.

### 3.4 Metal–CO Interactions

As we are mainly interested in catalytic applications of the high-level *ab initio* methods, we have set up a model system that comprises a transition metal (M) and a carbon monoxide interacting via the carbon atom (C–O bond distance has been fixed to the experimental value of 1.128 Å). The metallic center acts as the simplest representation of the CO binding active site in the catalyst. In these calculations, we have varied the M–C distance in order to obtain interaction curves with various methods. Such triatomic systems are small enough to permit reference CCSD(T) calculations, and testing can be performed with basis sets of various sizes. This set will be termed M–CO neutral. Because positively charged Lewis centers are often more reactive toward small molecule activation, we also propose another set of singly charged triatomics from the M–CO set. This set will be denoted as M–CO cation. For both sets, the metal selection includes catalytically relevant M = Au, Ni, Cu, Pd and Pt atoms. The platinum and platinum ion have been tested with two spin multiplicities, 1/3 and 2/4, respectively. The summary of metallic centers along with their spin multiplicities used in the calculations is provided in Table 5.

**Table 5:** Spin Multiplicities (Mult) of Metallic Centers Used in the M–CO Calculations

neutral	mult	cation	mult
Au	2	Au^+^	1
Ni	3	Ni^+^	4
Cu	2	Cu^+^	1
Pd	1	Pd^+^	2
Pt	1	Pt^+^	2
Pt	3	Pt^+^	4

Mean average errors (MAEs) of the interaction energies averaged over M–C distances of 1.6–2.6 Å are presented for neutral and cationic M–CO systems in Table 6 and Table 7, respectively. Such distances have been chosen to cover a range around the average M–C equilibrium bond length of the chosen transition metals. The tables provide results for basis sets of increasing size, from the compact def2-SVP up to extended def2-QZVPP. Interaction energies are calculated with respect to systems where M–C distance is set to a large value (10 Å).

**Table 6:** Correlation Energies Error Evaluation [in kcal/mol] of the Original (DLPNO) and Corrected (cDLPNO) DLPNO-CCSD(T0/T1) Methods against the Canonical CCSD(T) Values Obtained for the M–CO Neutral Systems, Provided in Table 5^a^

	NormalPNO				TightPNO					
	T0		T1		T0		T1			
	DLPNO	cDLPNO	DLPNO	cDLPNO	DLPNO	cDLPNO	DLPNO	cDLPNO	Extr.(5/6)^45^ T1(T0)	Extr.(6/7)^45^ T1(T0)
Def2-SVP										
Au	1.73	0.35	0.97	0.48	1.25	0.55	0.40	0.33	1.15(1.97)	0.40(0.59)
Cu	3.13	1.74	2.33	0.94	2.01	1.13	0.99	0.40	1.56(2.55)	0.33(1.28)
Ni	4.03	2.46	2.91	1.18	2.58	1.30	1.22	0.39	1.62(2.51)	0.38(1.67)
Pd	2.85	1.62	1.76	0.59	1.99	1.48	0.76	0.29	1.43(2.63)	0.25(1.31)
Pt(Mult = 1)	2.35	0.58	1.22	0.87	1.93	1.25	0.76	0.31	0.72(1.96)	0.19(1.33)
Pt(Mult = 3)	2.13	0.83	1.22	0.22	1.92	1.05	0.94	0.10	2.15(0.80)	0.30(1.77)
Def2-TZVP										
Au	2.64	1.27	1.77	0.42	2.12	1.52	1.16	0.56	1.43(2.43)	0.43(1.48)
Cu	3.32	1.60	2.42	0.78	2.64	1.64	1.57	0.57	2.13(3.00)	0.97(2.19)
Ni	3.85	2.22	2.97	1.33	2.67	1.94	1.51	0.79	2.51(3.46)	0.60(1.96)
Pd	3.39	1.73	2.05	0.54	2.58	1.66	1.10	0.30	1.33(2.87)	0.41(1.97)
Pt(Mult = 1)	3.10	1.37	1.73	0.71	2.74	2.06	1.37	0.70	0.66(2.24)	0.95(2.33)
Pt(Mult = 3)	3.39	1.94	2.46	1.00	2.70	2.06	1.67	1.03	4.28(4.87)	0.54(1.38)
Def2-TZVPP										
Au	2.61	1.19	1.78	0.36	1.96	1.03	1.02	0.12	1.81(2.71)	0.14(1.17)
Cu	3.18	1.23	2.25	0.38	2.50	1.01	1.39	0.26	1.47(2.44)	0.95(2.20)
Ni	3.75	1.83	2.83	0.90	2.68	1.58	1.60	0.60	1.85(2.81)	0.97(2.22)
Pd	2.98	1.59	1.59	0.37	2.58	1.60	1.11	0.23	0.83(2.41)	0.61(2.16)
Pt(Mult = 1)	2.39	0.58	0.97	1.02	2.48	1.50	1.13	0.44	1.44(2.89)	0.53(1.93)
Pt(Mult = 3)	2.81	1.69	1.91	0.79	2.24	1.51	1.25	0.52	3.99(4.56)	0.48(0.97)
Def2-QZVPP										
Au	2.79	1.29	2.01	0.51	2.06	0.99	1.18	0.12	1.96(2.77)	0.29(1.27)
Cu	3.14	1.02	2.30	0.40	2.42	0.75	1.42	0.42	2.94(3.78)	0.53(1.68)
Ni	3.50	1.72	2.70	0.91	2.67	1.40	1.69	0.53	2.67(3.56)	0.80(1.93)
Pd	2.85	1.16	1.53	0.30	2.34	1.46	0.91	0.12	1.26(2.71)	0.21(1.72)
Pt(Mult = 1)	2.71	1.12	1.34	0.61	2.02	0.79	0.54	0.80	0.73(1.82)	0.30(1.47)
Pt(Mult = 3)	2.83	1.47	1.99	0.64	2.36	1.43	1.45	0.52	2.82(4.96)	0.55(1.12)

a Extrapolated (6/7) values with the (T0) and (T1) corrections have been obtained by the procedure described by Altun and co-workers.^45^ Errors presented are the MAE averaged over 1.6–2.6 Å M–C distances of the respective PES curves.

**Table 7:** Correlation Energies Error Evaluation [in kcal/mol] of the Original (DLPNO) and Corrected (cDLPNO) DLPNO-CCSD(T0/T1) Methods against the Canonical CCSD(T) Values Obtained for the M–CO Cationic Systems, Provided in Table 5^a^

	NormalPNO				TightPNO					
	T0		T1		T0		T1			
	DLPNO	cDLPNO	DLPNO	cDLPNO	DLPNO	cDLPNO	DLPNO	cDLPNO	Extr.(5/6)^45^ T1(T0)	Extr.(6/7)^45^ T1(T0)
Def2-SVP										
Au^+^	1.65	0.18	1.10	0.43	1.21	0.46	0.59	0.17	1.50(1.97)	0.40(0.65)
Cu^+^	2.04	0.98	1.55	0.50	1.37	0.47	0.76	0.32	0.9(1.57)	0.31(0.97)
Ni^+^	0.21	0.89	0.42	0.84	0.68	1.10	0.68	0.33	1.37(1.08)	0.75(0.86)
Pd^+^	1.63	0.65	1.12	0.30	1.09	0.31	0.51	0.29	0.82(1.38)	0.12(0.72)
Pt^+^(Mult = 2)	0.76	0.19	0.25	0.57	0.35	0.62	0.33	1.18	1.11(0.46)	0.27(0.33)
Pt^+^(Mult = 4)	1.54	0.53	1.01	0.20	1.22	0.55	0.62	0.13	0.36(0.98)	0.27(0.93)
Def2-TZVP										
Au^+^	1.77	0.42	1.07	0.29	1.58	0.77	0.84	0.21	1.33(2.00)	0.40(1.07)
Cu^+^	2.08	0.72	1.41	0.23	1.67	0.83	0.93	0.10	1.24(1.97)	0.55(1.34)
Ni^+^	1.51	0.59	1.23	0.31	1.27	0.80	0.94	0.47	1.21(1.52)	0.54(0.91)
Pd^+^	1.75	0.43	1.16	0.17	1.29	0.45	0.64	0.20	0.15(0.85)	0.27(0.96)
Pt^+^(Mult = 2)	0.94	0.57	0.40	1.17	0.63	0.26	0.10	0.79	0.91(0.61)	0.20(0.44)
Pt^+^(Mult = 4)	2.09	0.73	1.48	0.21	1.56	0.96	0.88	0.28	0.79(1.51)	0.45(1.18)
Def2-TZVPP										
Au^+^	1.93	0.72	1.20	0.20	1.61	0.64	0.82	0.20	1.38(2.07)	0.41(1.12)
Cu^+^	2.05	0.85	1.37	0.20	1.62	0.64	0.84	0.15	0.88(1.63)	0.52(1.38)
Ni^+^	1.34	0.49	1.04	0.26	1.11	0.55	0.76	0.20	1.11(1.42)	0.35(0.76)
Pd^+^	1.84	0.68	1.20	0.19	1.46	0.59	0.78	0.15	0.94(1.66)	0.31(1.05)
Pt^+^(Mult = 2)	1.24	0.32	0.55	0.76	0.91	0.28	0.20	0.67	0.37(1.15)	0.22(0.58)
Pt^+^(Mult = 4)	2.03	0.91	1.42	0.37	1.55	0.91	0.87	0.23	1.42(2.10)	0.30(1.05)
Def2-QZVPP										
Au^+^	2.22	0.83	1.38	0.19	1.65	0.78	0.73	0.16	0.58(1.80)	0.23(1.12)
Cu^+^	2.10	0.69	1.38	0.13	1.69	0.98	0.88	0.25	1.00(1.78)	0.44(1.35)
Ni^+^	1.63	0.78	1.33	0.51	1.29	0.61	0.94	0.29	1.11(1.43)	0.63(1.01)
Pd^+^	2.03	0.72	1.37	0.18	1.73	0.87	0.99	0.17	1.30(2.03)	0.56(1.35)
Pt^+^(Mult = 2)	1.08	0.34	0.34	1.18	0.93	0.31	0.14	0.88	0.23(0.98)	0.32(0.62)
Pt^+^(Mult = 4)	2.10	0.75	1.48	0.32	1.79	0.70	1.10	0.19	1.23(1.90)	0.61(1.38)

a Extrapolated (6/7) values with the (T0) and (T1) corrections have been obtained by the procedure described by Altun and co-workers.^45^ Errors presented are the MAE averaged over 1.6–2.6 Å M–C distances of the respective PES curves.

In almost all cases, the proposed correction scheme reduced the DLPNO error by about 50%. The improvement is consistent for both the neutral and cationic species. One should note that the absolute errors for M–CO neutral are larger than those obtained for M–CO cationic. This is mainly due to the small energy gap between nd^*x*^(n + 1)s^*y*^ and nd^*x*–1^(*n* + 1)s^*y*+1^ electronic configurations of M atoms. For the same reason, low-spin Pt cationic systems show a small increase of the error when our correction is applied. We have noted that despite the same UHF reference, the MP2 and CCSD(T) unrelaxed densities display significantly different 5s occupations. In fact, this underlines the importance of the static correlation effects, which are not meant to be covered by the single-reference methods.

Our correction shows expected behavior with respect to the choice of triples treatment (T0 or T1): error obtained with the cDLPNO-CCSD(T1) scheme is generally lower than the corresponding cDLPNO-CCSD(T0) error. Some departure from this behavior is observed for the cationic Pt–CO system, e.g., error for the cDLPNO-CCSD(T1) is larger than that for the cDLPNO-CCSD(T0) by about 0.5 kcal/mol with the def2-SVP basis set and TightPNO settings. Similar deviations were observed for some of the extrapolated values, especially those obtained from (5/6) extrapolation (e.g., neutral Pt–CO at a multiplicity 3 with def2-SVP basis set). Again, this could be traced back to similar issues observed in the MOBH35 data set.^55,72^

**Figure 1:**
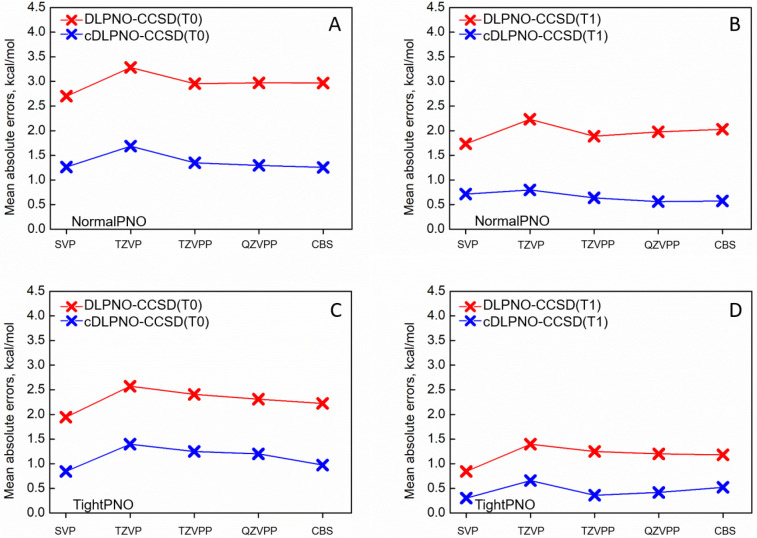
Averaged MAE obtained for the M–CO neutral PES profiles for the Def2-SVP, Def2-TZVP, Def2-TZVPP, Def2-QZVPP basis sets and for the complete-basis set limit (CBS),^73^ obtained with the 3/4 extrapolation. Red curves represent the original DLPNO errors, and blue curves depict the errors of the corrected energy values. Results obtained with the NormalPNO for the (T0) and (T1) triples are presented in panels A and B, respectively. Results obtained with the TightPNO for the (T0) and (T1) triples are presented in the panels C and D, respectively.

We have found that the proposed scheme provides a well-defined increase in the accuracy with minor dependence on the basis set size (Figure 1). This finding is of special importance because of the recently published result stating the dependence of the DLPNO error on this parameter.^37^ It is also evident that the use of (T1) instead of (T0) is mandatory to bring the calculations in the chemical accuracy regime (error <1 kcal/mol). Our correction scheme brings cDLPNO-CCSD(T1) TightPNO and NormalPNO calculations below this threshold for the tested triatomics.

## 4 Conclusions

The presented MP2-based correction scheme provides an economic, yet accurate, way to account for the PNO space truncation in the DLPNO-CCSD(T) calculations. Importantly, the method is free of any fitted parameters. We have shown that the proposed correction allows us to minimize the impact of the *T*_cutPNO_ choice on the accuracy of the results for both closed- and open-shell systems. Especially the latter can be thought of as a hard test since MP2 is known to fail often in this case.

Our approach displays remarkable robustness, but it cannot remove inherent DLPNO-CCSD(T) issues, like dynamic correlation-induced orbital relaxation effects. Special care should also be taken in cases where static correlation effects come into play, like near-degeneracies. In these cases, MP2 may describe a different electronic state than the DLPNO-CCSD(T) method and deteriorate results. For such systems, however, the applicability of single-reference methods should be carefully examined. In this study, we did not test any non-Hartree–Fock reference determinants, such as DFT orbitals. While these are not an issue with coupled cluster methods containing the singles operator, they become problematic at the MP2 level. The latter, in most implementations, assumes Brillouin’s theorem to hold, i.e., singles are not computed.

The current correction scheme can be extended to not only correct for the PNO space truncation but also estimate the DLPNO-CCSD(T) CBS limit with the MP2 method. In principle, DLPNO-MP2, canonical MP2, or even MP2-F12^74^ may be used for the basis set extrapolation. We are now examining the accuracy and efficiency of various choices to develop cDLPNO-CCSD(T)/CBS(δMP2) model chemistry, and this will be the scope of the upcoming work.

## Supporting Information Available

M–CO energetics and error analyses (ZIP)
Figures S1 and S2, example ORCA input file along with a compound job script, detailed energetics and error analyses for each benchmark set (XLSX)
